# Magnetic resonance imaging of the brachial plexus. Part 2: Traumatic injuries

**DOI:** 10.1016/j.ejro.2022.100397

**Published:** 2022-01-22

**Authors:** Pawel Szaro, Mats Geijer, Bogdan Ciszek, Aleksandra McGrath

**Affiliations:** a Department of Radiology, Institute of Clinical Sciences, Sahlgrenska Academy, University of Gothenburg, Gothenburg, Sweden; b Region Västra Götaland, Sahlgrenska University Hospital, Department of Radiology, Gothenburg, Sweden; c Department of Descriptive and Clinical Anatomy, Centre of Biostructure Research, Medical University of Warsaw Chałubinskiego 5, 02–004 Warsaw, Poland; d Department of Clinical Sciences, Lund University, Lund, Sweden; e Department of Neurosurgery, Bogdanowicz Memorial Hospital, Niekłanska 4/24, 03–924 Warsaw, Poland; f Umeå University, Faculty of Medicine, Department of Surgical and Perioperative Sciences, Umeå, Sweden; g Umeå University, Faculty of Medicine, Department of Clinical Sciences, Professional Development, Umeå, Sweden

**Keywords:** BP, brachial plexus, DT, diffusion tensor, MRP, multiplanar reformation, OBPP, obstetric brachial plexus palsy, STIR, short tau inversion recovery, TI, inversion time, TOS, thoracic outlet syndrome, TSE, turbo spin-echo, Brachial plexus, Injury, Brachial plexus surgery, Magnetic resonance imaging, Anatomy, Treatment

## Abstract

The most common indications for magnetic resonance imaging (MRI) of the brachial plexus (BP) are traumatic injuries. The role of MRI of the BP has increased because of recent trends favoring earlier surgery. Determining preganglionic vs. postganglionic injury is essential, as different treatment strategies are required. Thus, MRI of the BP should be supplemented with cervical spine MRI to assess the intradural part of the spinal nerves, including highly T2-weighted techniques. Acute preganglionic injuries usually manifest as various combinations of post-traumatic pseudomeningocele, the absence of roots, deformity of nerve root sleeves, displacement of the spinal cord, hemorrhage in the spinal canal, presence of scars in the spinal canal, denervation of the back muscles, and syrinx. Spinal nerve root absence is more specific than pseudomeningocele on MRI. Acute postganglionic injuries can present as lesions in continuity or tears. The following signs indicate injury to the BP: side-to-side difference, swelling, partial, or total BP rupture. Injury patterns and localization are associated with the mechanism of trauma, which implies a significant role for MRI in the work-up of patients.

The identification and description of traumatic lesions involving the brachial plexus need to be systematic and detailed. Using an appropriate MRI protocol, obtaining details about the injury, applying a systematic anatomical approach, and correlating imaging findings to relevant clinical data to make a correct diagnosis. Information about the presence or suspicion of root avulsion should always be provided.

## Highlights

• Trauma is the most common indication for MRI of the brachial plexus.
• MRI of the brachial plexus should include cervical spine MRI.
• Spinal nerve root absence is seen in preganglionic injuries.
• Determining preganglionic vs. postganglionic injury is essential for treatment planning.
• Appropriate MRI rapport is crucial in communication with the clinician.

## 1 Introduction

The current article is focused on diagnostic imaging with magnetic resonance (MRI) and its role in diagnosing common BP traumatic injuries from the perspectives of both radiologists and surgeons. Non-traumatic lesions with anatomical considerations and MR techniques are covered in Part 1 (Magnetic resonance imaging of the brachial plexus. Part 1: Anatomical considerations, magnetic resonance techniques, and non-traumatic lesions).

The importance of MRI of the BP has been growing, fueled by an increased number of clinical indications and new trends in the early surgical treatment of BP injuries [1]. The increased interest in imaging of the BP has resulted in better MRI protocols with higher image quality. However, the wide choice of protocols can be confusing and time-consuming for radiologists and clinicians; thus, a consensus of which protocol to use is valuable. The description and reporting of the brachial plexus's pathologies need to be systematic and detailed (Table 1, Table 2).

**Table 1:** Diagnostic pearls to consider while evaluating a brachial plexus magnetic resonance study.

Diagnostic pearls
The side-to-side difference is relevant - therefore, it is worth examining the other side with a large field-of-view.
The increased signal on T2-weighted or STIR sequences are best evaluated on coronal sections; it is important to adjust the scan plane to the course of the BP.
Fat stranding is not visible in trauma while preserved in TOS, thus it is useful to include a T1-weighted sequence without fat suppression.
Evaluation of vessels is recommended because pseudoaneurysm or dissection may be a manifestation of vascular trauma (thrombosis or rupture).
Fractures or dislocations correlate with CT, so it is essential to evaluate any rib fractures that may be significant.

**Table 2:** Checklist for the radiological report for trauma/obstetric brachial plexus palsy cases.

Checklist for the radiological report for trauma/obstetric brachial plexus palsy cases
*MRI of the right/left brachial plexus* Is there a root avulsion? Which spinal nerves are involved? Is it a total or partial injury? Is there a meningocele? Are there rootlets traversing the myelomeningocele?
*Trunks, divisions, and cords* Which structures are injured? Is there a distal shift of the brachial plexus? What is the distance between parts of the ruptured plexus? Is there a hematoma? Is there a fracture of the clavicle or ribs? If a chest examination has been performed, are there any signs of the phrenic nerve being affected?

This review article aims to present the most common traumatic pathologies of the brachial plexus in adults and children from clinical and radiological perspectives.

## 2 Indications and aim of diagnostic imaging in the context of surgical treatment

The aim of the assessment of BP after trauma is to determine the localization of the injury, whether the injury is preganglionic or postganglionic, as different treatment strategies are required for the respective injuries. A preganglionic injury (root avulsion) disrupts the connection between the spinal nerve and spinal cord, while a postganglionic injury is located within the peripheral nervous system and does not disrupt the connection with the central nervous system. No spontaneous recovery is possible in root avulsion, while in postganglionic injury, depending on the severity, patients can often obtain useful function without surgical intervention. The traditional approach to adult BP injuries was to "wait and see" for 3–6 months to determine whether spontaneous recovery occurred. There has been a shift in strategy because of both experimental and clinical studies and systematic reviews suggesting the beneficial effect of early surgery [1], [2]. After early surgery, better results might be related to avoiding, in part, motoneuron death in preganglionic injuries [3], [4]. Brachial plexus exploration after a longer delay is also technically demanding because of the massive scar enveloping the nerves. Ultra-early exploration within two weeks after injury, when hematoma is still present, and the components of the BP are easily identified and dissected, is an attractive concept. Studies suggest that reconstruction of the BP within two months significantly improves muscle function recovery [5], [6]. Early exploration is a new and promising trend that has been adopted by several centers, leading to increased demands on MRI imaging. Nerve conduction studies and electromyography (EMG) do not usually give reliable results until three weeks after injury. Waiting until root avulsion is shown to be present by EMG is considered unnecessary by some surgeons and likely to worsen the patients' outcome [2], [7], [8], [9], [10].

MRI assessment of traumatic pathologies of the brachial plexus (BP) is a reliable tool with high sensitivity (93%) and specificity (72%) [11]. Indications for performing a BP MRI for trauma are broad and not fully defined, ranging from adult traumatic injuries, such as high or low-energy injuries with stretching of the nerves or a neck injury associated with weakness of the upper limb muscles and paresthesia on the same side, to obstetric BP palsy (OBPP) that is present immediately after birth with weakness or lack of shoulder abduction, elbow flexion and a flail upper limb in some cases [6].

### 2.1 Clinical diagnosis of brachial plexus injuries

A correct diagnosis of BP pathology is based on an appropriate clinical examination. Nerve conduction studies, EMG, MRI, and computed tomography (CT) myelography are complementary [3], [5], [12], [13]. Traditionally, nerve conduction studies and EMG, which can first be performed three weeks after the injury, have played an essential role in traumatic injuries and helped determine whether the damage is preganglionic or more postganglionic. However, in a modern setting where earlier surgery is preferred, their role decreases.

### 2.2 Traumatic disorders of the brachial plexus

#### 2.2.1 Traumatic brachial plexus injury

**Fig. 1:**
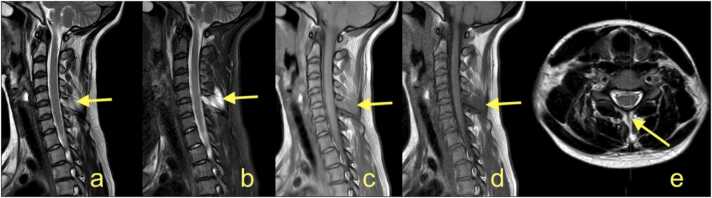
The cervical spine magnetic resonance imaging protocol used at the authors' institution is included in the examination protocol of the brachial plexus. (a) T2-weighted turbo spin-echo (TSE) sequence, (b) short tau inversion-recovery (STIR) sequence, (c) proton density-weighted TSE sequence, (d) T1-weighted TSE sequence, and (e) T2-weighted TSE sequence. A 24-year-old patient was involved in a car accident. There was complete paralysis of the C4-C8 segments, partially of the T1 segment in the motor fibers. MRI showed a rupture of the interspinous ligament (arrow) and the ligament flavum. Continued in Fig. 2.

**Fig. 2:**
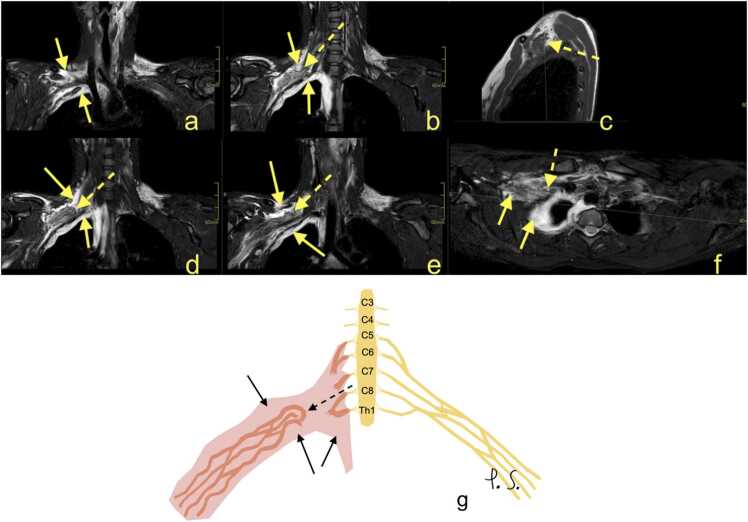
Continuation from Fig. 1. Magnetic resonance of the brachial plexus (BP). (a, b, d-f) T2-weighted modified DIXON sequence, (c) T1-weighted turbo spin-echo sequence. A right-sided hematoma was seen directly adjacent to the brachial plexus and in the right pleura (arrows). There was a total rupture of the BP trunks, with retraction and wrapping of the stumps of the BP (dashed arrows). G – the schematic drawing corresponding to magnetic resonance imaging (description as above).

The most common indications for MRI are traumatic injuries (Figs. 1 and 2) [5], [10], [13]. Recent reports indicate that BP stretching or laceration constitutes about 50% of BP trauma [10], [14], [15], [16]. Older reports often cite "the law of 70%" [17] which, while not entirely accurate, is a valuable mnemonic for radiologists. It states that 70% of BP injuries are caused by motor vehicles, of which 70% of accidents involve a bicycle or motorcycle, of which 70% have associated injuries. The 70% with multiple injuries have a supraclavicular trauma, of which 70% have root avulsion, with 70% of avulsions located at the levels C7-Th1 or C8-Th1 [14], [17]. However, avulsion of the nerve roots C5, C6, and C7 happen due to distraction, which can occur in the coronal place when a cyclist or motorcyclist falls on the ground having their head forcibly flexed to the opposite side. Avulsion of the nerve roots C8 and Th1 may occur when the arm is abducted over the head and force is applied simultaneously on the arm and trunk, e.g., when falling from a tree holding on to the branch [17]. Fig. 2.

**Fig. 3:**
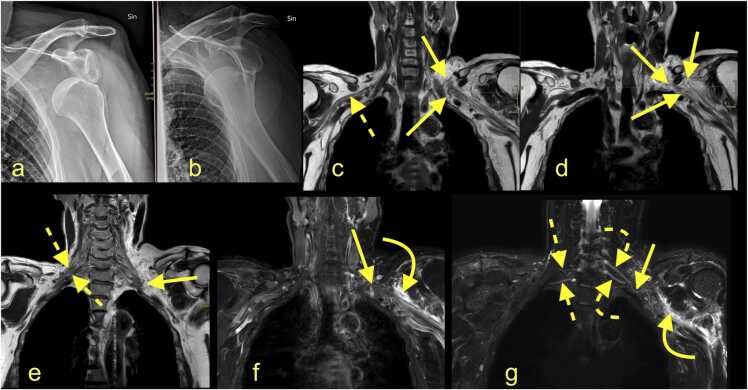
(a and b) A 43-year-old patient presented with an anterior shoulder dislocation on the left, which was quickly repositioned. Before repositioning, there was no function in the left elbow and hand. Magnetic resonance imaging of the left brachial plexus (BP) with (c-e) T2-weighted DIXON sequence, (f) T2-weighted DIXON, sequence with fat suppression, and (g) neurography showed total rupture of the left brachial plexus at the level of the cords. The stumps are significantly frayed (arrows), and the proximal stumps are thickened (curved dashed arrows). The area of rupture is surrounded by hematoma (curved arrows). The normal BP is on the right side (dashed arrows). Continued in Fig. 4.

**Fig. 4:**
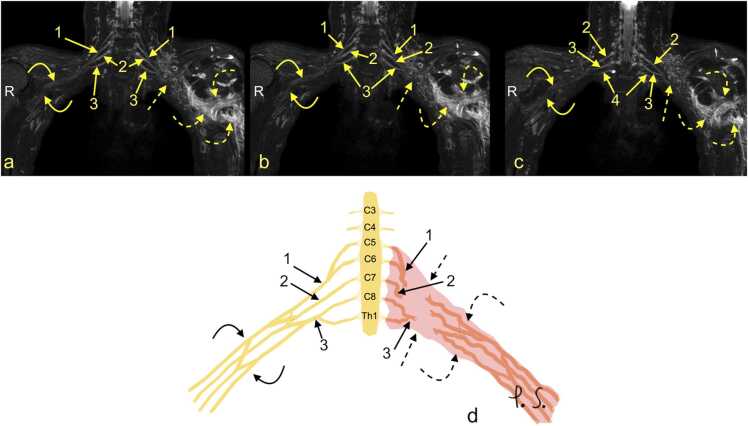
Continuation from Fig. 3. (a-c) Magnetic resonance neurography. Total rupture of the left brachial plexus (BP; dashed arrows) is accompanied by hematoma in the axillary cavity (curved dashed arrows). The right BP is normal (curved arrows). 1 – the upper trunk, 2 – the middle trunk, 3 – the C8 root, and 4 – the Th1 root. D – the schematic drawing corresponding to magnetic resonance imaging (description as above).

Two main risk groups might sustain BP injury. Individuals younger than 40 years involved in traffic accidents often have more severe injuries, while patients over 40 years with a history of anterior shoulder dislocation or humeral fracture often have axillary nerve involvement with poor prognosis (Figs. 3 and 4). In younger patients with a surgical neck fracture of the humerus or anterior shoulder dislocation, the outcome is often better because of transient motor nerve paralysis [10], [12].

In a suggested MRI protocol suitable for trauma patients, sequences in three planes are acquired to cover the entire BP (Fig. 1, Fig. 2). Contrast is not administered routinely; however, contrast enhancement may indicate the functional impairment of a nerve despite its continuation [18]. This examination is supplemented with a cervical spine MRI to assess the intradural part of the spinal nerves (Fig. 1).

There are other possible MRI protocols of the BP; more recently published papers show the high accuracy of highly T2-weighted techniques [19], [20], [21], [22]. However, no consensus on the most optimal MRI protocol exists [5], [10], [14], [20], [23], [24]. Some authors also recommend CT myelography as a gold standard for evaluating nerve root avulsion [18].

With the modern trend to perform early surgery, MRI should be performed shortly after injury. Appropriate interpretation may be challenging because of extensive adjacent post-traumatic changes in the soft tissues [14], [15]. Muscular edema may be revealed 24 h after nerve injury, probably due to the vascular dilation related to nerve trauma.

Acute preganglionic injuries usually manifest as various combinations of post-traumatic pseudomeningocele, the absence of roots, deformity of nerve root sleeves, displacement of the spinal cord, hemorrhage in the spinal canal, presence of scars in the spinal canal, denervation of the back muscles, and a syrinx [14], [20], [23], [25]. A preganglionic injury requires nerve transfer surgery [14], [24], while postganglionic injuries are treated by nerve grafting (with additional nerve transfers) or are followed up in cases of partial injury [18]. A chronic preganglionic injury may manifest as pseudomeningocele; however, most of the cases of pseudomeningocele are not related to the previous injury. Thus, the root absence sign is more specific [14], [18], [20]. Some suggest that pseudomeningoceles are an unreliable indicator of root avulsion [11]. Involvement of the interscalene space is significant because it can indicate injury to the BP roots. Fig. 4.

**Fig. 5:**
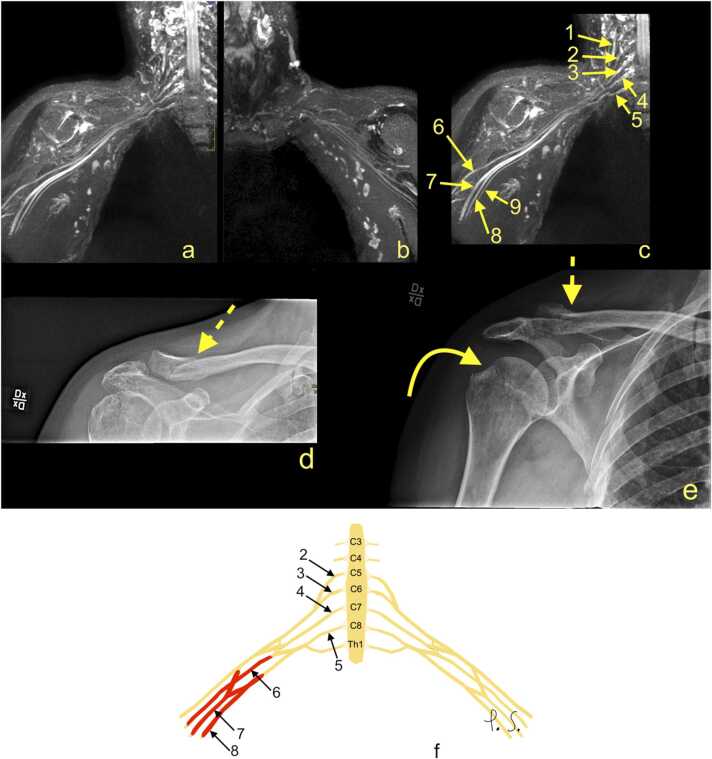
(a-c) Magnetic resonance imaging (MRI) of the brachial plexus (BP) of a 55-year-old patient involved in a car accident three months earlier. There is reduced strength in the right forearm and hand muscles, mainly in the muscles supplied by the median nerve. Radiating pain from the axillary cavity towards the thumb, index, and middle fingers on the right side were reported. (a) MRI showed a more prominent BP on the right side, mainly regarding the median and ulnar nerves compared to (b) the left BP. (d and e) Radiographs obtained three months before MRI showed a fracture of the acromial end of the right clavicle (dashed arrow) and fracture of the right tuberculum majus (curved arrow). 1 – the contribution from C4 to the BP, 2 – the C5 root, 3 – the C6 root, 4 – the C7 root, 5 – the C8 root, 6 – the radial nerve, 7 – the median nerve, 8 – the ulnar nerve, and 9 – the medial cutaneous nerve of the forearm. F – the schematic drawing corresponding to magnetic resonance imaging (description as above).

Acute postganglionic injuries can present as lesions in continuity (neuroma in continuity) or tears. The following signs may be seen: side-to-side difference (Fig. 5), swelling, partial or total BP rupture [18]. The radiological report should emphasize the total rupture of one component or retraction of the entire BP. Chronic postganglionic injuries usually present as scar tissue, fibrosis, or neuromas [23].

**Fig. 6:**
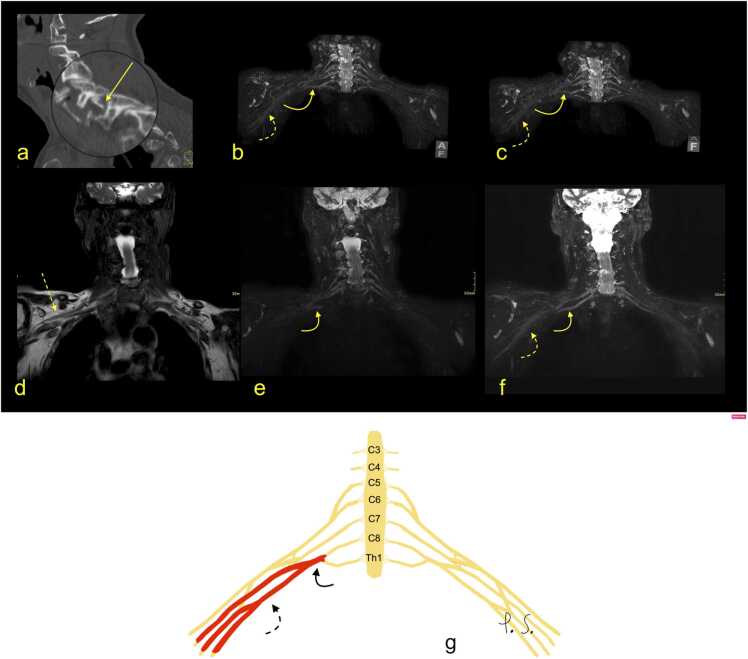
(a) An 83-year-old patient suffered from a fracture of the superior articular process C7 on the right side two months ago (arrow), after a fall from a height of 1 m, possibly with an armrest caught in his axilla. After the injury, he experienced a gradual deterioration of neurological function in the right arm, triceps atrophy, and loss of wrist extension and ability to grasp with increasing pain in the C7 area and allodynia and normal reflexes. Nerve conduction studies and EMG suggested a brachial plexus (BP) injury. (a) Computed tomography shows an unstable fracture of the articular facet with subluxation. (b-f) Magnetic resonance imaging revealed edema in the lower trunk of the BP (curved arrow) and slight edema in cords (dashed curved arrow). There was no hematoma adjacent to the right BP (dashed arrow). G – the schematic drawing corresponding to magnetic resonance imaging (description as above).

**Fig. 7:**
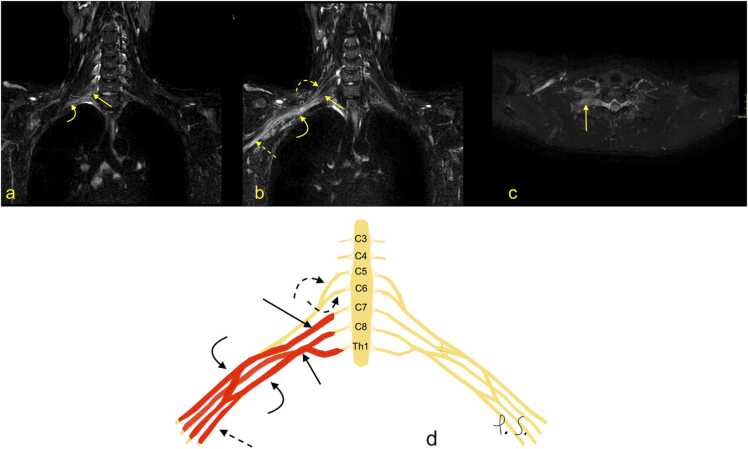
A 27-year-old patient was involved in a motocross accident three weeks earlier. Computed tomography of the whole body did not show any cervical spine or shoulder fracture. The patient experienced a loss of function in the right forearm and hand with diminished sensibility in the area supplied by the ulnar nerve and loss of finger abduction and opposition of the thumb. Clinically, brachial plexus (BP) injury was suspected. (a-c) MR neurography revealed a side-to-side difference, with diffuse edema of the right BP starting in the middle and lower trunks (arrows) and involving the cords (curved arrow) and their branches (dashed arrow). Notice the normal signal and structure of the C5 and C6 roots (curved dashed arrow). D – the schematic drawing corresponding to magnetic resonance imaging (description as above).

**Fig. 8:**
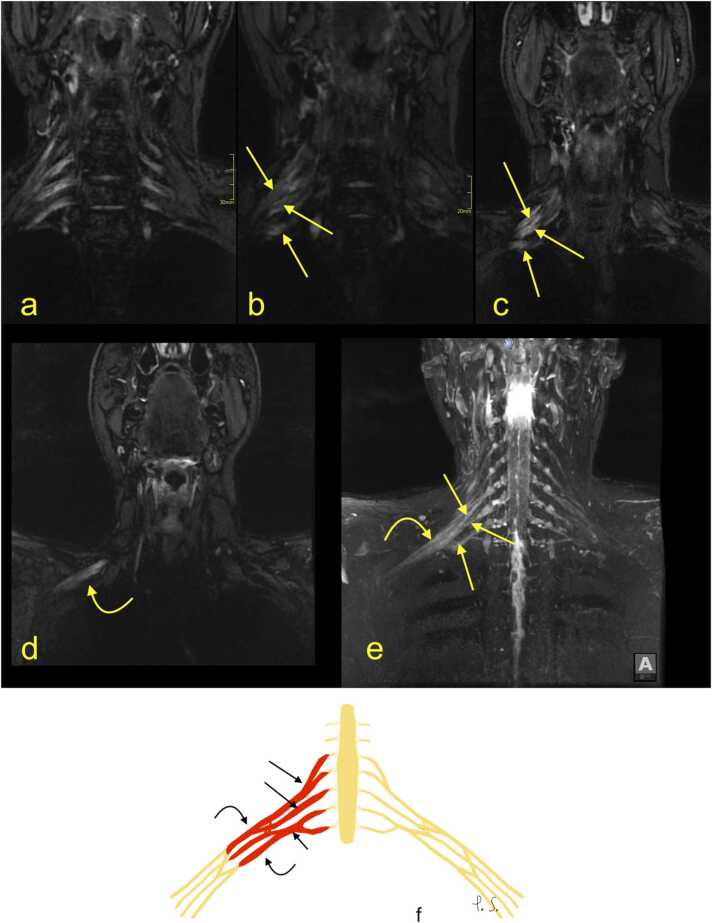
A 22-year-old patient was involved in a car accident two months earlier without fracture or dislocation. The patient presented with paresthesia of various degrees in the upper limb, weakness of the hand muscles, and pain in the arm and forearm. Clinically, the symptoms emanated from the C5-Th1 segments. MRI with (a-d) STIR and STIR MIP-reformation showed side-to-side difference and diffuse edema of the trunks (arrow) and cords (curved arrow). F – the schematic drawing corresponding to magnetic resonance imaging (description as above).

**Fig. 9:**
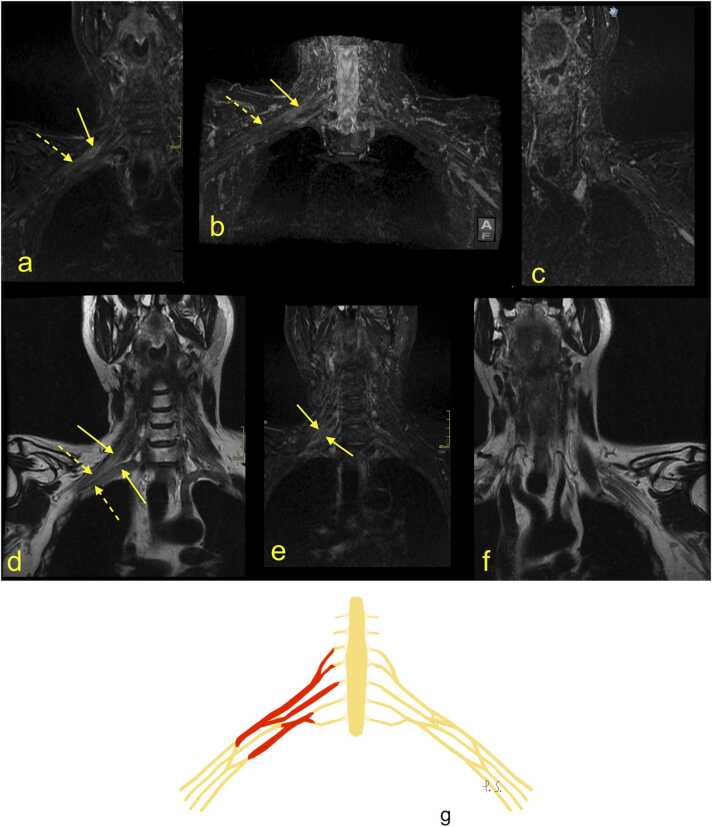
A 50-year-old patient fell from a height of 2 m, after which he experienced tingling and numbness in the right forearm and hand and weakness of the muscles of the upper limb, mainly in the forearm and hand. Nerve conduction studies and electromyography showed brachial plexus (BP) injury. Clinically, the symptoms suggested injury of the median, ulnar, and radial nerves. (a-f) G – the schematic drawing corresponding to magnetic resonance imaging of the BP (description as above). Magnetic resonance imaging of the BP showed edema of the trunks (arrow) and cords (dashed arrow). There was no side-to-side difference in the proximal parts of the long nerves.

**Fig. 10:**
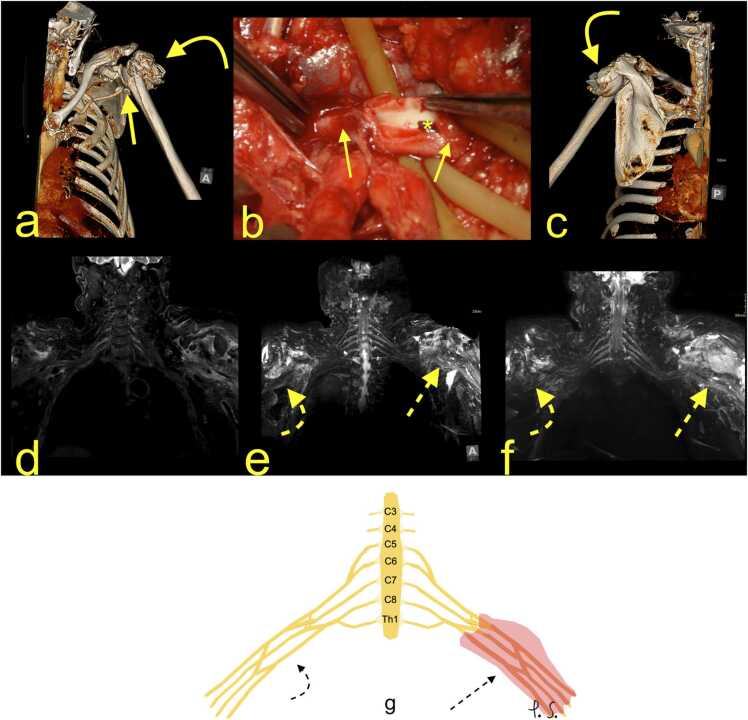
A 75-year-old patient fell down 18 steps of a staircase. (a, c) Whole-body CT showed a comminuted proximal humerus fracture with displacement (curved arrow) and dissection of the left axillary artery (arrow). (b) Surgical correlation from the emergency surgery, which restored flow in the axillary artery (* – a clot in the artery). The patient lacked motor function in the muscles supplied by the radial, median and ulnar nerves. Clinically, the diagnosis was the left brachial plexus (BP) injury. (d-f) Magnetic resonance imaging showed significant side-to-side asymmetry with edema of the infraclavicular part of the BP on the left at the level of the cords and nerves. There is hematoma in the soft tissue around the fracture (dashed arrow). The right BP is normal (curved dashed arrow). G – the schematic drawing corresponding to magnetic resonance imaging (description as above).

MRI signs of BP injury without rupture may manifest as edema of the nerves (Fig. 6, Fig. 7, Fig. 8, Fig. 9), which in the MR image is expressed as BP asymmetry. Often, BP injury is related to fractures of the cervical spine (Fig. 6) or trauma without fracture (Fig. 7, Fig. 8, Fig. 9). More severe shoulder girdle injuries may result in the dissection of the axillary artery (Fig. 10). The restoration of blood flow in the axillary artery often determines the clinical outcome. CT angiography allows the site and degree of dissection of the axillary artery to be determined (Fig. 10). The presence of a hematoma related directly to the BP suggests injury. The MR scan shows fluid or fluid collection bands adjacent to the BP. Changes in muscle signal may follow the BP injury. The acute denervation of the muscles is visible as a higher signal on MRI, followed by muscle atrophy and fatty degeneration.

Injury patterns and localization are associated with the mechanism of trauma. The most common injury site is the supraclavicular part of the BP in blunt trauma [9], while penetrating gunshot injuries frequently affect the infraclavicular part of the BP [6]. The roots and trunks of the BP are more prone to injury than the divisions or fascicles. Injury of the infraclavicular part of the BP can also be associated with injury to the shoulder region, such as anterior shoulder dislocation or proximal humerus fracture. In general, the most common type of injury is a total rupture of roots C5-Th1 (about 75%), followed by upper plexus, C5-C6 injury (about 20%), and less commonly, lower plexus, C8-Th1 (about 3%) [8].

### 2.3 MRI correlation to microscopic injury

The higher the degree of nerve damage, the worse the regeneration potential and more significant indication for nerve reconstruction [16], [18], [25]. Higher grades of nerve injury may cause muscle abnormalities, which are manifestations of denervation. An increased MR signal of the nerve without an increase in diameter or change in muscle signal indicates neuropraxia, which corresponds to myelin injury (Grade 1). Increased signal and edema of the nerve combined with the higher signal from the muscles indicate axonotmesis (Grade 2), which corresponds to myelin and axon fiber injury (Fig. 3). The increased signal from the swollen nerve corresponds to a discontinuity of the axon and endoneurium with intact perineurium and epineurium (Grade 3). Focal enlargement and heterogeneous nerve signals may indicate neuroma in continuity because of the injury of microscopic structure within the nerve, except for the epineurium (Grade 4). Higher signals of an intact nerve on MRI with abnormal muscle signal correspond to a grade 2–4 injury (Fig. 6, Fig. 7, Fig. 8, Fig. 9). Total nerve discontinuity presents as hemorrhage or fibrosis in the gap with epineural thickening (Grade 5) (Fig. 1, Fig. 2, Fig. 3, Fig. 4). A combination of different injury grades across the nerve is frequently seen (Grade 6) [25].

### 2.4 Obstetric brachial plexus palsy (OBPP)

Obstetric BP palsy (OBPP) due to traction injury to the upper extremity nerves occurs in 1.5–5.1/1000 births [7], [26], [27]. OBPP is most likely a traction injury associated with certain risk factors such as maternal diabetes, shoulder dystocia, high fetal weight, and breech presentation. However, there are known cases of OBPP occurring after Cesarean section, which raises concerns that OBPP might also have an intrauterine cause. OBPP most often affects the upper or total plexus and less frequently only the lower plexus (lower BP palsy is often associated with breech presentation). The child presents with varying degrees of weakness of the shoulder, elbow, wrist, and in cases of total BP injury, hand function. Like adults, a child with root avulsion and T1 involvement will present with Horner's sign.

**Fig. 11:**
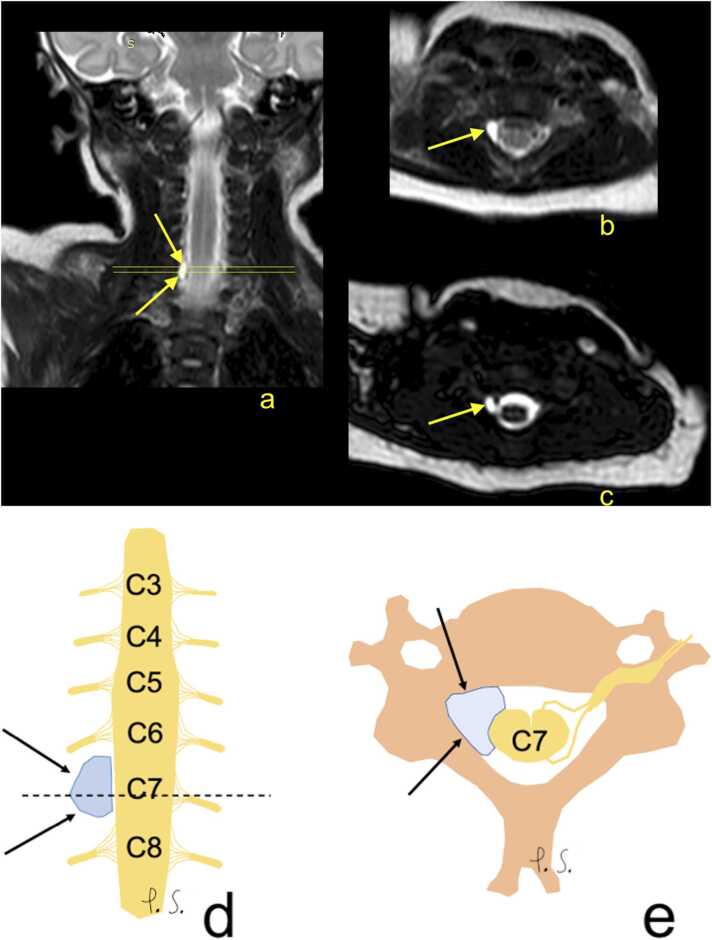
A 5-week-old girl with Erb's palsy on the right side was caused by a birth injury. The paresis was visible from birth, according to the mother, with slightly improved function after a time. The child was born at 40 weeks with a birth weight of 3730 g and 10 points on the Apgar scale. Radiography of the clavicle and thorax showed no post-traumatic changes. (a, b, c) T2-weighted images showed pseudomeningocele (arrow) at the level of C6/C7 on the right side with suspicion of a right C7 root avulsion, which was confirmed at surgery. D and e- schematic drawings corresponding to magnetic resonance imaging, d corresponds to 11a while e corresponds to 11 b and c (description as above).

Identifying the presence of root avulsion is a vital goal to guide reconstruction (Fig. 11). The widely accepted timing of OBPP surgery at present is 2–3 months for total BP injury and 3–6 months for upper/lower plexus injury. During the surgery, the BP is explored, and the extent of the injury is confirmed intraoperatively. Similar to surgery of the ruptured adult BP, the surgical strategy involves connecting both nerve stumps with a nerve graft. In avulsion injuries with insufficient proximal nerves connected to distal nerves, nerve transfer is performed, "borrowing" a healthy nerve to connect to an injured distal nerve. MRI is considered the gold standard for the diagnosis of OBPP. The role of other diagnostic examinations, such as preoperative nerve conduction and electromyography, is controversial because of low sensitivity in identifying preganglionic ruptures [28].

One of the potential donors for nerve transfer in pediatric patients is the intercostal nerve (usually from the upper intercostal spaces). Therefore, it is essential to alert the surgeon to coexisting rib fractures (causing potential injury to intercostal nerves) or elevated diaphragm pointing to phrenic nerve involvement (which can cause respiratory insufficiency postoperatively if intercostal nerves are used for nerve transfer at the same time). Similar to adults, pseudomeningocele is not a definite sign of root avulsion. However, its presence suggests avulsion, especially in the absence of visible rootlets traversing the pseudomeningocele [29].

There are specific anatomical considerations for neonatal plexus MRI (Fig. 11). The BP in the supraclavicular fossa has less vertical direction than in adult patients and passes more transversely and laterally, which requires changes to the imaging planes. The subclavian artery takes a similar pathway, and the relation of the lower trunk of the BP to the subclavian artery is more posterior rather than inferior compared to adults. The adult MRI protocol should not be directly applied to infants because of differences in the BP direction in the specific patient. Three-dimensional MRI may assess avulsion spinal nerve roots (Fig. 11) [30], [31], [32].

## 3 Summary

The identification and description of pathologies involving the brachial plexus need to be systematic and detailed. Using an appropriate MRI protocol, obtaining details about the injury, applying a systematic anatomical approach, and correlating imaging findings to relevant clinical data to make a correct diagnosis.

### Funding

This project received no funding.

### Ethics approval and consent to participate

Not applicable. No ethics approval is required for this educational review.

### Consent for publication

Not applicable. The manuscript does not contain the data of individuals in any form.

### Authors' contributions

**PS** conceived the idea of the pictorial review to publish this study. **PS** selected appropriate MRI figures and prepared the schematic drawings. **PS, MG, BC, AMG** analyzed and annotated the chosen images. **PS, AMG, and MG** wrote the first draft of the manuscript. All authors read and approved the final manuscript.

## Declaration of Competing Interest

The authors declare that they have no known competing financial interests or personal relationships that could have appeared to influence the work reported in this paper.

## Data Availability

Yes.
